# Contribution of Travelers to Plasmodium Vivax Malaria in South West Delhi, India: Cross-Sectional Survey

**DOI:** 10.2196/50058

**Published:** 2025-01-08

**Authors:** Deepali Savargaonkar, Bina Srivastava, Chander Prakash Yadav, Mrigendra Pal Singh, Anup Anvikar, Amit Sharma, Himmat Singh, Abhinav Sinha

**Affiliations:** 1 ICMR-National Institute of Malaria Research, Sector 8, Dwarka, New Delhi, 110077, India, 91 9205059972; 2 ICMR-National Institute of Cancer Prevention and Research, Noida, India; 3 ICMR-National Institute for Research in Tribal Health, Jabalpur, India; 4 Academy of Scientific and Innovative Research, Ghaziabad, India; 5 International Centre for Genetic Engineering and Biotechnology, New Delhi, India

**Keywords:** malaria, Plasmodium vivax, imported malaria, population movement, transmission, elimination, India

## Abstract

**Background:**

India is committed to malaria elimination by the year 2030. According to the classification of malaria endemicity, the National Capital Territory of Delhi falls under category 1, with an annual parasite incidence of <1, and was targeted for elimination by 2022. Among others, population movement across states is one of the key challenges for malaria control, as it can result in imported malaria, thus introducing local transmission in an area nearing elimination.

**Objective:**

This descriptive study attempts to assess the contribution of such imported *Plasmodium vivax* cases to the malaria burden in South West Delhi (SWD).

**Methods:**

A cross-sectional study was carried out at the fever clinic of the Indian Council of Medical Research-National Institute of Malaria Research in SWD from January 2017 to December 2019. Demographic and travel history data were recorded for all *P vivax* confirmed malaria cases diagnosed at the fever clinic. Vector and fever surveys along with reactive case detection were conducted in SWD and Bulandshahr district of Uttar Pradesh, 1 of the 6 geographical sources for a high number of imported malaria cases.

**Results:**

A total of 355 *P vivax* malaria cases were reported during the study period. The proportion of imported cases was 63% (n=222). Of these, 96% (n=213) of cases were from Uttar Pradesh. The distribution of malaria cases revealed that imported cases were significantly associated with travel during the transmission season compared with that in the nontransmission season. Entomological and fever surveys and reactive case detection carried out in areas visited by imported *P vivax* malaria cases showed the presence of adults and larvae of *Anopheles* species and *P vivax* parasitemia.

**Conclusions:**

Population movement is a key challenge for malaria elimination. Although additional *P vivax* infections and vector mosquitoes were detected at places visited by the imported malaria cases, the inability to detect the parasite in mosquitoes and the possibility of relapses associated with *P vivax* limit the significance of malaria associated with the travel. However, there remains a need to address migration malaria to prevent the introduction and re-establishment of malaria in areas with very low or 0 indigenous cases.

## Introduction

Malaria is a parasitic disease transmitted by the bites of infected female *Anopheles* mosquitoes. The estimated number of malaria cases worldwide in 2021 was 247 million spread over 84 countries with 619,000 deaths. Although the World Health Organization (WHO) South East Asia region comprised ≈2% of the estimated global burden, India contributed to ≈79% of these cases, with a preponderance of *Plasmodium vivax* over *Plasmodium falciparum* [1].

Migration, both international and within-country, is a recognized social health determinant of multiple diseases across the globe, and malaria is no exception. Various factors put the migrating population at risk of contracting malaria, and these include their socioeconomic, living, working, and transit conditions [2]. The risk of malaria is also to the host communities that provide shelter to the migrants, particularly when the migration is along an epidemiological gradient from a high-burden to a low-burden or nonendemic area, putting malaria elimination efforts at risk [3-5]. Several pieces of evidence of this have been documented in the context of international migration [6-8] and within-country migration [9,10], including that in India [11].

India is committed to malaria elimination by 2030 and has formulated the National Framework for Malaria Elimination that classifies Indian states and union territories into 4 categories from 0 to 3, with category 3 being the highest-burden areas with an annual parasite incidence (API) of ≥1 per thousand persons at risk [12]. To achieve the elimination goal in the desired time frame, special focus needs to be given to the identified challenges by the National Center for Vector Borne Diseases Control (NCVBDC). Population size and migration are recognized as important challenges for malaria elimination, apart from asymptomatic parasite reservoirs, low-density infections, and parasite- and vector-resistance [13-15]. The movement of populations across and within Indian states is one of the key challenges in malaria control [12], and particularly, the migration of workers in large numbers from rural areas to cities has been reported in India [16]. Similarly, malaria cases among the mobile population contribute to a high percentage of total malaria cases in many countries [17] and have been a recognized challenge for malaria elimination [18]. Hence, it is important to carry out the screening and treatment for malaria in mobile populations for control and elimination of malaria in endemic areas, especially National Framework for Malaria Elimination category 1 areas (with API<1), and for prevention of the re-establishment of local transmission of malaria [12] in areas that have eliminated malaria (category 0 with 0 indigenous cases).

Despite being the capital of India that attracted >100,000 migrants each year since 2012 [19], no study on malaria in the migrant population in Delhi has yet been reported. Delhi falls under category 1 (API<1 per 1000 persons at risk), and its neighboring state, Uttar Pradesh (UP), is in category 2 with an API of less than 1 but with some districts having an API of 1 or more. With such a magnitude of migration, there remains a sustained threat of the introduction of *Plasmodium* infection by infected migrant populations from high-burden areas to the areas in categories 0 and 1 [16].

As Delhi and its neighboring states have *P vivax* as the predominant *Plasmodium* species causing malaria, this study was therefore carried out to assess the contribution of imported *P vivax* cases to the *P vivax* malaria burden in South West Delhi (SWD) by tracking the travel history of infected patients diagnosed at the fever clinic of Indian Council of Medical Research-National Institute of Malaria Research (ICMR-NIMR). Additionally, the study also aimed to identify mosquito breeding habitats and the presence and types of mosquito species in areas where these imported malaria cases resided/visited, and to detect additional *Plasmodium* infections/malaria cases through reactive case detection (RACD) and fever surveys in selected areas visited by these patients. These entomological and fever surveys and RACD were done to further identify any ongoing transmission in the areas visited by patients with imported malaria to support the hypothesis that these are indeed the cases likely to import malaria from areas with ongoing transmission to SWD.

## Methods

### Ethical Considerations

The study was approved by the Institutional Ethics Committee of ICMR-NIMR (ECR/NIMR/EC/2015/507 and ECR/NIMR/EC/2019/175). Informed consent was obtained from all human participants who were involved in the study. The participant identifier data were anonymized. No compensation was provided to the participants.

### Study Sites, Samples, and Definitions

The study was initiated at the fever clinic at ICMR-NIMR, SWD, and later expanded to include the prominent catchment areas of the fever clinic of SWD and 6 villages of 1 selected district (Bulandshahr) of UP. Incoming febrile patients were screened for *Plasmodium* infection at the fever clinic of ICMR-NIMR from January 2017 to December 2019. Basic demographic data including age, gender, history of fever, and travel details (if any) were recorded using a paper-based structured questionnaire. The parasitological diagnosis at the clinic was performed by microscopy. Thick and thin blood smears were prepared, stained using the Jaswant Singh–Bhattacharji stain [20] and examined under 100× magnification. Microscopy was performed independently by 2 trained microscopists. In case of a discrepancy, a third trained microscopist examined the smears, and consensus observation by 2 trained microscopists was considered final. Those diagnosed with malaria were treated as per the national drug policy [21].

History of travel during the preceding 30 days of fever, including the places visited and duration of stay, was verbally elicited through a calendar-based recall method among all patients with malaria. Obtained travel history was used to classify the patients into 3 nonoverlapping categories: no travel history (patients neither traveled in or out of their residence in SWD), returning travelers (patients who were residing in SWD and traveled outside Delhi but came back), and incoming travelers (patients who were not residing in SWD but transiently traveled to SWD). Returning travelers were further classified based on the duration of stay outside Delhi into those returning to SWD within 7 days and those returning between 7 and 30 days. Similarly, incoming travelers were also classified into those who came to SWD within and beyond 30 days of fever.

Since the incubation period for malaria is 7 to 30 days [22-25], returning travelers with malaria who returned to SWD between 7 and 30 days of fever onset and incoming travelers who entered SWD within 30 days of fever onset were classified as imported malaria cases (acquired infection outside Delhi) for this study. The rest of the patients with malaria were considered to have indigenous infections.

Imported malaria cases were further investigated for the exact village and district of travel based on their recorded travel history. Thus, to further investigate whether the malaria cases were imported or indigenous, malaria vector (anopheline) surveys were carried out both in SWD and in the villages the imported malaria cases traveled to. These surveys were carried out by field workers adequately trained in entomology in catchment areas (Raj Nagar and Bagdola; every month from September 2018 to December 2019) of the fever clinic of ICMR-NIMR predominantly reporting malaria and also in villages (once in October 2019) of Bulandshahr district of UP state. Fever surveys and RACD were also carried out in these villages of Bulandshahr, UP. Bulandshahr district was preferred out of the 6 districts that showed significant sources of imported malaria in UP based on the burden of imported cases, logistic convenience, and operational feasibility. All 6 villages of Bulandhahr districts that had epidemiologically relevant travel connections with the imported malaria cases were surveyed.

The vector survey included the collection of mosquitoes (adults and larvae) from the houses of reported cases and their surrounding houses, species identification, and enumeration of mosquito breeding habitats. Resting adult mosquito collection was conducted in households of 6 villages of Bulandshahr district during early morning (6 AM to 8 AM) using hand aspirators. The larval collection was also done in each village from all water-bearing sites, that is, ponds, ditches, large cement tanks, drains, and seepages, in peridomestic and domestic areas of each village. In the Raj Nagar catchment locality of SWD, the houses were searched from 7 AM to 9 AM, and larval collection was also conducted simultaneously if searched houses and containers were found positive for larval presence from domestic and peridomestic water bodies and other sites including overhead tanks, large open water bodies, tires, coolers, bird pots, flowerpots, iron containers, and solid wastes in urban catchment areas of the clinic and from domestic and peridomestic containers in the houses of reported cases. The collected larvae were reared in an insectary separately up to their emergence to identify the mosquito species. Identification of species was done following the standard taxonomic key as described by Christophers [26]. Adult mosquito collection was also done using the hand catch method [27], and the collected *Anopheles* mosquitoes were screened for the presence of malaria parasites (*P falciparum, P vivax, Plasmodium malariae*, and *Plasmodium ovale*), through polymerase chain reaction (PCR) [28], in pools of mosquitoes made village-wise and species-wise. To estimate the critical density of malaria vectors, per man-hour density (PMHD) was calculated as the number of anophelines collected per hour by an insect collector using the formula:


$$PMHD=\frac{No.\;of\;mosquitoes\;collected}{Time\;spent\;(in\;hours)\;x\;no.\;of\;insect\;collectors}$$

Fever surveys and RACD were carried out in October 2019 in 6 villages of the Bulandshahr (UP) with support from the local health personnel (Accredited Social Health Activists and Health Inspectors). Fever camps were organized at a central location in each village, and the local health personnel informed the villagers about the camp and motivated them to visit. Incoming febrile cases were screened for malaria by using a rapid diagnostic test (SD Bio Line Malaria Ag P.f / P.v, Standard Diagnostics, Inc, Republic of Korea) as per the manufacturer’s instructions. All febrile cases were treated symptomatically, and patients with malaria were treated as per the national drug policy. RACD was done as described by the WHO [29], and blood smears were prepared from apparently healthy individuals in and around the household of the index cases. The smears were examined for the presence of malaria parasites at ICMR-NIMR, Delhi (as described previously), and the results were communicated to the concerned health personnel for further management.

### Data Entry and Statistical Analysis

All the collected data were entered in a Microsoft Excel 2016 spreadsheet and presented as proportions (percentages), medians, and ranges, where appropriate. The strength of association was estimated using a chi-square test, and a *P* value of less than .05 was considered statistically significant.

## Results

### Overview

A total of 14,748 fever cases were screened for malaria by microscopy from January 2017 to December 2019. The 3-year period prevalence of malaria was 2.4% (364/14,748). Out of these 364 cases, 355 (97.5%) were *P vivax* mono-infections, 8 *P falciparum* mono-infections (2.1%), and 1 mixed infection of *P vivax* and *P falciparum*. There was male predominance among patients with fever (59%) as well as patients with *P vivax* malaria (71%). More than half of the patients with *P vivax* malaria were in the age group of 15‐29 years (183/355, 52%) with a median age of 22 years. The parasite burden ranged from 63 to 206,187 parasites per microliter of blood.

### Imported Malaria Burden

**Figure 1.**
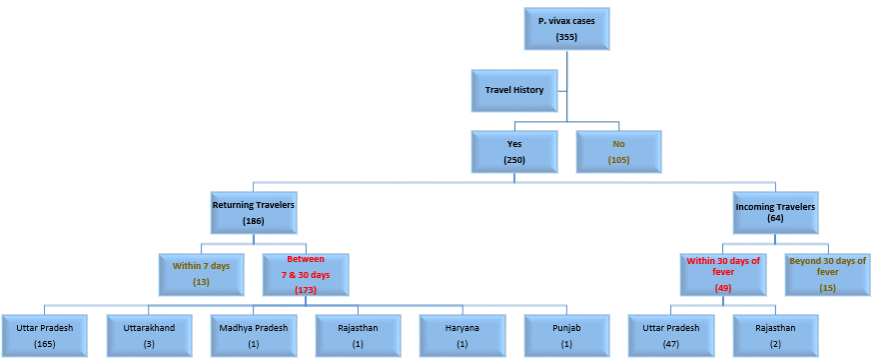
Travel history among 355 patients with *Plasmodium vivax* malaria. Based on the epidemiologically relevant travel history, cases associated with travel were categorized into “imported” (222; shown in red font) and indigenous (133; gold colored font) cases. The geographical distribution (states) of imported cases is also mentioned.

**Figure 2.**
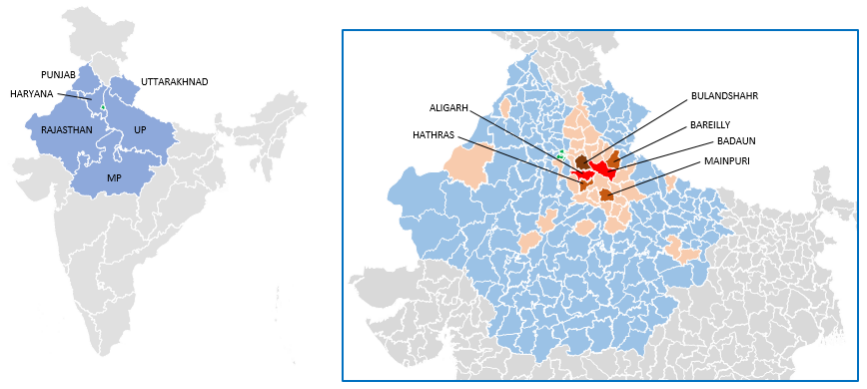
Areas traveled to by *Plasmodium vivax* malaria imported cases. The areas (states and union territories of India) are shown in blue (Punjab, Chandigarh, Haryana, Uttarakhand, UP, MP, and Rajasthan) whereas Delhi (study site) is shown in green. The zoomed-in image of the map in the inset shows further administrative breakdown of these 7 states and union territories (as districts) in blue. The districts within these 7 states and union territories, which are associated with the travel history of imported cases, are colored based on the number of imported cases contributed by each district: light orange (1‐5 cases); dark orange (5‐15 cases); darker orange (15‐25 cases), and red (>25 cases). It is evident that UP has 3 dark orange districts: Hathras (10 cases), Mainpuri (12 cases), and Bareilly (15 cases); 1 darker orange district: Bulandshahr (25 cases); and 2 red districts: Aligarh (37 cases) and Badaun (47 cases). MP: Madhya Pradesh; UP: Uttar Pradesh.

Out of the 250 *P vivax* cases with a travel history (250/355, 70%), 186 (74%) cases were returning travelers and the remaining 64 (26%) cases were incoming travelers (Figure 1). However, relevant travel history to be able to label them as imported cases was available from 63% (222/355) of patients. Out of these imported *P vivax* cases, 173 (78%) cases were returning and 49 (22%) cases were incoming travelers. Ninety-five percent (212/222) of the imported cases were from UP and 142 of them (142/212, 67%) had traveled to 1 of the 6 districts of UP viz Bareilly, Badaun, Aligarh, Hathras, Bulandshahr, and Mainpuri. The remaining 10 imported cases had traveled to Uttarakhand (3; Dehradun and Hardwar), Rajasthan (3; Bikaner, Bundi, and Sawai Madhopur), Madhya Pradesh (2; Gwalior), Haryana (1; Gurugram), and Punjab (1; Sri Muktsar Sahib), as shown in Figure 2. Out of 9 patients with *P falciparum* malaria (including 1 mixed infection), 8 had a travel history.

The reasons for travel in *P vivax* malaria cases included visiting their native residence in various states (mainly for returning travelers); education or employment (for incoming travelers); and visiting relatives, family, and friends during festivals (for returning and incoming travelers) as many Indian and regional festivals temporally coincide with the malaria transmission season. The minimum length of stay outside Delhi among travelers was 1 day while the maximum stay was of 111 days. Travel history was reported by the family members of the patients with malaria as well. There were 24 families with at least 2 members probably acquiring malaria after traveling.

A majority of the *P vivax* cases (279/355, 79%) were detected during the transmission season, that is, July to November. The nontransmission season (December to June) contributed to the remaining 21% (76/355) of cases.

**Figure 3.**
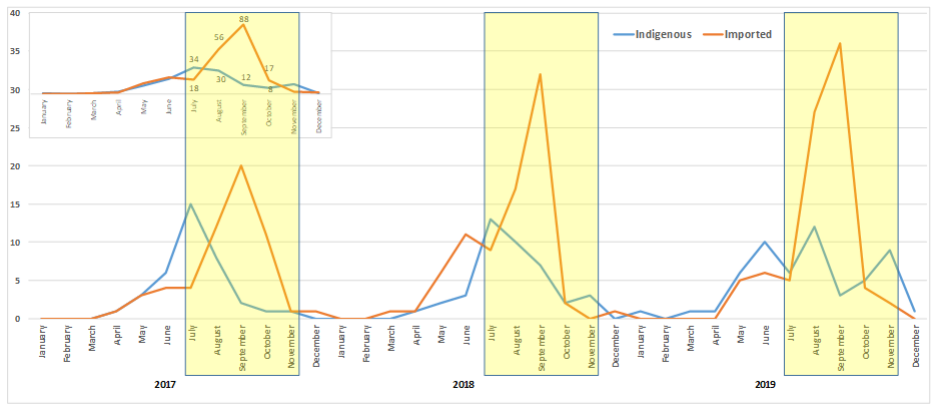
*Plasmodium vivax* malaria cases reported in Delhi (2017‐2019). The figure shows the number of *P vivax* cases, indigenous (blue) and imported (imported), as reported each month and each year during the study period (January 2017 to December 2019) at the Indian Council of Medical Research-National Institute of Malaria Research fever clinic. The cumulative month-wise data from 2017 to 2019 are shown in the inset. The yellow semitransparent rectangles show the transmission season.

During the transmission season (July to November; 2017‐2019), the proportion of imported *P vivax* malaria cases diagnosed at the fever clinic was higher (65%) than that in the nontransmission season (53%; December to June), as shown in Figure 3, and the difference was statistically significant (*χ*^2^_1_=4.04; *P*=.031) at a 95% confidence level.

### Entomological Surveys

Adult mosquito collections from the draining and catchment areas (Bagdola and Raj Nagar areas) of the fever clinic in SWD showed 4 mosquito species, with a low prevalence of *Anopheles* mosquitoes during the survey (September 2018 to December 2019). Out of 573 adult mosquitoes collected from 3395 houses, only 7 (1%) were *Anopheles stephensi* (Table 1), and all of them were found during the malaria transmission season (September). No other species of *Anopheles* were found. The majority of mosquitoes (443/573, 78%) were *Aedes aegypti* in the households.

**Table 1.** Month-wise adult mosquito collection in Bagdola and Raj Nagar, South West Delhi, between September 2018 and December 2019. Transmission season is highlighted in gold. The proportion of mosquito species identified out of the total is shown as a percentage (in parentheses).

		Houses checked, n	Mosquito species identified				
			*Aedes aegypti*, n (%)	*Aedes albopictus*, n (%)	*Anopheles stephensi*, n (%)	*Culex quinquefasciatus*, n (%)	Total, n
Month and year							
	September 2018^a^	316	28 (37)	0 (0)	1 (1)	46 (62)	75
	October 2018^a^	97	14 (70)	0 (0)	0 (0)	6 (30)	20
	November 2018^a^	402	63 (73)	0 (0)	0 (0)	23 (27)	86
	December 2018	220	16 (84)	1 (5)	0 (0)	2 (11)	19
	January 2019	112	1 (100)	0 (0)	0 (0)	0 (0)	1
	February 2019	70	0 (0)	0 (0)	0 (0)	0 (0)	0
	March 2019	179	2 (40)	0 (0)	0 (0)	3 (60)	5
	April 2019	60	13 (100)	0 (0)	0 (0)	0 (0)	13
	May 2019	344	10 (63)	0 (0)	0 (0)	6 (37)	16
	June 2019	367	89 (87)	0 (0)	0 (0)	13 (13)	102
	July 2019^a^	352	53 (75)	0 (0)	0 (0)	18 (25)	71
	August 2019^a^	270	61 (91)	0 (0)	6 (9)	0 (0)	67
	September 2019^a^	301	44 (90)	5 (10)	0 (0)	0 (0)	49
	October 2019^a^	211	40 (100)	0 (0)	0 (0)	0 (0)	40
	November 2019^a^	33	9 (100)	0 (0)	0 (0)	0 (0)	9
	December 2019	61	0 (0)	0 (0)	0 (0)	0 (0)	0
Total		3395	443 (78)	6 (1)	7 (1)	117 (20)	573

a Transmission season.

On the other hand, a total of 9 species of mosquitoes (5 anopheline, 2 *Aedes*, 1 *Culex,* and 1 *Armigeres* species) were collected from 6 villages in the Bulandshahr district (Table 2) of UP. Out of the 416 adult mosquitoes collected, 126 (30%) were *Anopheles* with *Anopheles subpictus* (21%) and *Anopheles culicifacies* (5%) dominating the anopheline burden. *Culex quinquefasciatus* was the major mosquito species found in rural Bulandshahr (235/416, 57%). Among the anophelines, *A subpictus* (PMHD 16.61) was the most abundant species followed by the main rural vector *A culicifacies* (PMHD 4.25) and *A stephensi* (PMHD 1.35).

**Table 2.** Adult mosquito collection and species identification in the surveyed 6 villages of Bulandshahr district of Uttar Pradesh during October 2019. The proportion of different mosquito species identified out of the total is shown as a percentage (in parentheses).

		Mosquito species identified									
		*Aedes aegypti*, n (%)	*Aedes alboipictus*, n (%)	*Anopheles stephensi*, n (%)	*Anopheles annularis*, n (%)	*Anopheles culicifacies*, n (%)	*Anopheles nigerrimus*, n (%)	*Anopheles subpictus*, n (%)	*Culex quinquefasciatus*, n (%)	*Armigeris subalbatus*, n (%)	Total, n
Villages											
	Adauli	1 (0.7)	0 (0)	0 (0)	1 (0.7)	9 (7)	4 (3)	30 (22)	86 (63)	6 (4)	137
	Lakhaoti	0 (0)	1 (2)	0 (0)	4 (6)	5 (8)	0 (0)	12 (18)	26 (40)	18 (27)	66
	Shikarpur (Kot Kalan 1)	4 (6)	2 (3)	5 (8)	0 (0)	4 (6)	0 (0)	6 (9)	45 (68)	0 (0)	66
	Kutubpur	0 (0)	0 (0)	0 (0)	1 (4)	0 (0)	0 (0)	5 (19)	20 (77)	0 (0)	26
	Mustafabad Daduwa	0 (0)	0 (0)	2 (5)	0 (0)	2 (5)	1 (2)	0 (0)	18 (41)	21 (47)	44
	Dinoul	0 (0)	0 (0)	0 (0)	0 (0)	2 (3)	0 (0)	33 (43)	40 (52)	2 (3)	77
Total		5 (1)	3 (0.7)	7 (2)	6 (1)	22 (5)	5 (1)	86 (21)	235 (57)	47 (11)	416

A total of 24 collected anopheline mosquito pools (22 mosquitoes in 6 pools of *A culicifacies*, 7 mosquitoes in 2 pools of *A stephensi,* 3 mosquitoes in 3 pools of *Anopheles annularis*, 86 mosquitoes in 11 pools of *A subpictus,* and 5 mosquitoes in 2 pools of *Anopheles nigerrimus*) were tested by PCR for the presence of malaria parasites; however, none of the pools was found positive for the presence of malaria parasites.

The vector survey to identify mosquito breeding habitats in Raj Nagar and Bagdola catchment localities revealed that out of 14,333 containers (including large containers, cemented tanks, and underground tanks) checked, *Anopheles* breeding was found only in 8 containers that included coolers, overhead tanks, cement tanks, and iron containers. There were no large water bodies in the surrounding area of the survey.

A mosquito breeding habitat survey in the 6 villages of Bulandshahr found that out of the 203 water-holding containers and water bodies, 51 (25%) had *Anopheles* breeding. Major breeding sites included drains (4/7, 57%), canals (1/2, 50%), ponds (3/6, 50%), and pits (9/20, 45%). Other sites where breeding was found included domestic and peridomestic water bodies (26/123, 21%), cemented ground tanks (cattle tanks; 5/26, 19%), and rice fields (3/19, 16%). *A culicifacies* were found mostly in canal banks and village ponds whereas *C quinquefasciatus* in sewages.

### Fever Surveys

Camp-based fever surveys in the 6 villages identified 86 persons with fever, with only 1 person testing positive for *P vivax* by the rapid diagnostic tests. RACD from 22 asymptomatic persons around the households of the index cases revealed 5 additional cases (5/22, 23%) of *P vivax* by microscopy.

## Discussion

### Principal Findings

Between January 2017 and December 2019, 355 monoinfected *P vivax* cases were reported, and out of them, 63% (n=223) could be categorized as possible imported malaria cases based on relevant travel history, thus forming a major burden of reported malaria cases in SWD. The study also detected 5 additional *P vivax* cases through RACD done in villages visited by the imported cases and identified malaria vectors of anopheline species and their breeding habitats in such areas.

The distribution of malaria cases reported in the fever clinic at ICMR-NIMR revealed that the malaria cases were more likely to be imported than indigenous and occur in transmission season. The period July to November is considered to be the malaria transmission season in Delhi, while December to June is considered a nontransmission season [30].

Although 67% of the *P vivax* cases were imported, being associated with relevant travel history, the remaining 37% of indigenous cases could be associated with possible local transmission of *P vivax* in SWD, as suggested by the presence of anophelines in Delhi (this study) and the reported presence of malaria vectors in Delhi [31,32]. *P vivax* malaria cases during the nontransmission season or in nontravelers might also be recurrences or relapses due to the activation of hypnozoites from the liver [30].

Recent travel within the country is associated with malaria in various studies [22,23]. This study showed that the proportion of males was more than females among imported as well as indigenous malaria cases and a similar trend was seen in patients with fever as well. In similar studies, men traveling away from home in the last 30 days were reported to be strongly associated with malaria in Ethiopia [24,25].

Although Delhi shares its borders with the state of UP in the east and the state of Haryana in the remaining directions, we observed that ≈96% of the imported cases were from UP. Data highlights of the census of India in 2001 and 2011 show that Delhi receives a higher number of migrants (≈50% of the total in-migrants) from UP versus that from Haryana (≈10% of in-migrants) [33]. With >20-fold higher malaria burden in UP (than in Haryana), the findings of >95% of cases being imported from UP are explainable [12]. Reasons for migration to Delhi are cited to be due to employment, business, education, marriage, etc [33,34]. The reasons for travel reported during this study were festivals, farming, and visits to relatives. Those visiting friends and relatives in malaria-endemic areas have been reported to be at high risk of contracting malaria [35,36].

Many districts in UP contributed to the imported *P vivax* cases in SWD (Figure 2); however, 6 UP districts contributed 10 or more cases: Hathras (10 cases), Mainpuri (12 cases), Bareilly (15 cases), Bulandshahr (25 cases), Aligarh (37 cases), and Badaun (47 cases). Further investigations (vector and fever surveys) were carried out in 6 villages of Bulandshahr district only due to reasons explained earlier. Bulandshahr district of UP, located southeast of Delhi, is situated between the Ganga and Jamuna rivers, which are the major rivers in North India. The soil is very fertile where mainly sugarcane, and wheat are grown. Irrigation facilities are also well-developed and this area is canal-irrigated as well [37] which makes the area highly mosquitogenic.

During vector surveillance in 6 villages of Bulandshahr, 25% of the water bodies were positive for anopheline larval breeding, and 5 species of adult *Anopheles* mosquito were found during adult mosquito collections. Unlike Bulandshahr, where almost every village had ponds, canals, and ample water in surrounding areas providing sufficient opportunities for the breeding of anophelines, Delhi is highly urbanized and lands are not available for ponds and crop fields. In comparison to Bulandshahr, the catchment areas of the fever clinic (Raj Nagar and Bagdola localities) of SWD had a very low prevalence of *Anopheles*. Only 1 species, that is, *A stephensi* was present in these localities in contrast to Bulandshahr where 5 species of *Anopheles* were collected out of which 2 were major malaria vectors, that is, *A stephensi* and *A culicifacies*. Larval surveys suggested that urban and rural areas have different breeding habitats. In villages, natural water bodies like ponds, canals, pits, and crop fields were more prominent and harbored more breeding than the peridomestic and domesticated containers in contrast to the urban areas where natural breeding sites are limited and were confined to peridomestic and domestic containers only. Mosquito species like *A stephensi* and *A aegypti* are adapted to breed in such urban areas whereas *A culicifacies* mostly breed in outdoor natural water habitats like canal banks, village ponds, etc and *C quinquefasciatus* is found in sewage water.

Among malaria vectors, *A culicifacies* was found to be the dominating mosquito species along with an efficient malaria-transmitting vector, *A stephensi*. However, the month of the survey (October) had low vector density, which may be due to the low ambient temperature (20‐25 °C) during the survey period. Further, the mosquitoes that were collected from the villages of Bulandshahr district did not show parasite positivity by PCR. This may be due to multiple factors, including the very short period of vector survey (30 d), a limited number of vectors collected toward the end of transmission (October), and the difference in time of mosquito collection and case reporting in the clinic, as the vector survey was carried out as a response for tracking ongoing transmission in areas previously visited by imported malaria cases.

The 6 districts of UP that contributed most to imported cases in SWD had API (2018) of 0.06 (Bulandshahr and Mainpuri), 0.1 (Hathras and Aligarh), 5.5 (Badaun), and 7.3 (Bareilly), whereas the API of Delhi during this period was 0.02 [38]. A survey was therefore, carried out in Bulandshahr wherein 1 out of 86 febrile cases (fever survey) and 5 out of 22 afebrile persons (RACD) were identified with *P vivax* infections which signifies that further studies are needed to assess the extent of asymptomatic *Plasmodium* infection and its role in transmission in such areas.

The prevalence of malaria was found lower in the camp-based fever surveys compared with the prevalence reported from the fever clinic in SWD. This may be because the camp-based fever surveys were carried out during October, which marks the end of the transmission season and therefore may have had a lower number of cases. Further, the catchment area of the camp included a village whereas the fever clinic at SWD has a much larger and densely populated catchment area.

The regions nearing malaria elimination tend to have a heterogeneous endemicity, with foci of high burden and areas with no endogenous malaria transmission. For eliminable diseases such as malaria, within-country migration is a recognized but understudied challenge in such geographically heterogeneous transmission to sustain zero-burden and prevent reintroduction and re-establishment of transmission [23,39]. Such regions often lack a robust surveillance system to deal with imported cases besides treating them, and there appears to be a lack of documented cross-reporting and targeted intervention in the foci where the infections probably originated.

This study is therefore important, as it attempted to comprehensively investigate imported malaria cases, and may be adopted and locally adapted as an implementation model in similar areas with no or few locally acquired malaria cases.

### Limitations

There is an obvious limitation of this study that a limited geographical area for fever and vector surveillance was selected. Nevertheless, the study shows the presence of malaria transmission in areas where patients with malaria reporting to the fever clinic had traveled. The study was also limited by the possibility of recall bias of study participants correctly recalling the exact dates of travel for both imported and indigenous patients with malaria. The investigators, however, tried to extract the near-exact dates by relating travel to the locally relevant cultural events, festivals, and other contextual events. Misclassification bias (incorrect classification of imported malaria) could have stemmed from the possibilities of recurrences and relapses of *P vivax* infections acquired before the study period. The study did not use available molecular methods to differentiate recurrent versus new infection and therefore could not account for *P vivax* relapses. However, the possibility that only up to 40% of *P vivax* infections in the study area (that too in the nontransmission season) could be due to possible relapses [30], nonavailability of molecular methods to confidently differentiate recurring infections from new infections, and random possibilities of recurrence in both the imported and indigenous patients with malaria may have compensated for this limitation.

Last, only 2 methods for mosquito collection were used. The hand catch method using an aspirator was the only method adopted for the estimation of vector density. For larval collection, dips were taken from water bodies for assessment of breeding. No other method was adopted for mosquito collection, and this might have underestimated the frequency of vectors and their possible infection with *Plasmodium*, because the PCR results did not show any vector positivity.

### Conclusions

A significant burden (63%) of *P vivax* malaria reported in SWD was found to be imported from UP. Malaria transmission possibilities (multiple breeding sites suggesting stable breeding ground of anophelines) were higher in Bulandshahr than in SWD. Indigenous cases in SWD are also a concern, as adult vectors were also found in the area. Despite the detection of additional *P vivax* cases following RACD in Bulandshahr and vector-breeding sites being identified, the conclusion that the imported cases really acquired infections from the surveyed areas in Bulandshahr is limited by the correct recall of travel or fever dates and the possibility of relapses due to *P vivax*.

### Way Forward

The study reiterates that population movement is a key challenge for malaria elimination, particularly in areas with very low or 0 indigenous malaria cases, and investigations of the potential role of travelers in introducing malaria and its further spread are definitely needed. Since the epidemiology of migration malaria is contextual, appropriate tailor-made measures are needed, both at the sites where imported cases are detected and in areas where these infections might have been acquired. In addition, effective “information, education, and communication” activities to educate travelers regarding the potential risks of travel-associated malaria and its prevention should be undertaken.

## Abbreviations

API: annual parasite incidence
ICMR-NIMR: Indian Council of Medical Research-National Institute of Malaria Research
NCVBDC: National Center for Vector Borne Diseases Control
PCR: polymerase chain reaction
PMHD: per man-hour density
RACD: reactive case detection
SWD: South West Delhi
SWD: South West Delhi
UP: Uttar Pradesh
WHO: World Health Organization
